# Supplementary material for: Quorum sensing modulates bacterial virulence and colonization dynamics of the gastrointestinal pathogen Citrobacter rodentium
Source: Gut Microbes. 2023 Oct 16;15(2):2267189. doi: 10.1080/19490976.2023.2267189 (PMC10580866; doi:10.1080/19490976.2023.2267189)

*Supplemental Material for:*

Quorum Sensing Modulates Bacterial Virulence and Colonization Dynamics of the Gastrointestinal Pathogen *Citrobacter rodentium*

## Jorge Peña-Díaz^1,2^, Sarah E. Woodward^1,2^, Anna Creus-Cuadros^1,2^, Antonio Serapio-Palacios^1,2^, Stephanie Ortiz-Jiménez^4^, Wanyin Deng^2^, and B. Brett Finlay^1,2,3,*^

## ^1^ Department of Microbiology and Immunology, University of British Columbia, Vancouver, BC, V6T 1Z3, Canada

## ^2^ Michael Smith Laboratories, University of British Columbia, Vancouver, BC, V6T 1Z4, Canada

## ^3^ Department of Biochemistry and Molecular Biology, University of British Columbia, Vancouver, BC, V6T 1Z3, Canada

## ^4^ Departamento de Microbiología Molecular, Instituto de Biotecnología, Universidad Nacional Autónoma de México, Cuernavaca, Morelos, México

## * Correspondence: [bfinlay@interchange.ubc.ca](mailto:bfinlay@interchange.ubc.ca)

Supplemental Figures and Tables:

**Table S1:** Bacterial strains used in this study.

| **Strain** | **Description** | **Source** |
| --- | --- | --- |
| DBS100 | *C. rodentium* ATCC 51459 | Schauer and Falkow^1^ |
| DBS100 ∆*croI* | Acyl-homoserine-lactone synthase mutant | (Croxen and Koymans,  unpublished) |
| DBS100 ∆*croR* | LuxR-type AHL receptor mutant | This study |
| DBS100 ∆*croI::croI* | Chromosomal complementation of ∆*croI* | This study |
| DBS100 ∆*croR::croR* | Chromosomal complementation of ∆*croR* | This study |
| DBS100 ∆*croI∆croR* | Acyl-homoserine-lactone synthase and LuxR-type AHL receptor mutant | This study |
| MFD*pir* | *E. coli* conjugal donor for biparental matings (DAP auxotroph) | Ferrie`res et al.^2^ |
| pTNS2 | *E. coli* with a Tn7 transposase expression plasmid (Amp*^R^*) | Choi and Schweizer^3^ |

**Table S2:** Plasmids used in this study.

| **Plasmids** | **Description** | **Source** |
| --- | --- | --- |
| pRE112 | Suicide vector for allelic exchange (Cam*R*) | Edwards et al.^4^ |
| pUC18R6KT-  mini-Tn7T | Mini-Tn7 base vector used for  constructing chromosomally complemented strains (Amp*^R^*) | Choi and Schweizer ^3^ |

**Table S3:** Oligonucleotide primers used for construction of mutant strains.

| **Primer Name** | **Sequence*** | **Notes** | |
| --- | --- | --- | --- |
| ∆croI1_FW | GTACGGTACCGAAGCTCTTGCGCCAT | KpnI | |
| ∆croI1_RV | GTACGCATGCTTCTCTTGTCAGGCGG | SphI | |
| ∆croI2_FW | ATGAACTCTGTTGTTGAGTTTCAACAA |  | |
| ∆croI2_RV | AAACTCAACAACAGAGTTCATTG |  | |
| ∆croR1_FW | aagcggtgtaagtgaactgcatgaattcccgggagagctcACATA GGTGGGTTTAGCATACTGGAAGATGATGGG | SacI | |
| ∆croR1_RV | GGGGCTCAGTTATCCAGTAAGTACGTATCCTT CACGTCAGTTACAGCCT |  | |
| ∆croR2_FW | CTGACGTGAAGGATACGTACTTACTGGATAA CTGAGCCCCGGATACCTGC |  | |
| ∆croR2_RV | ggcccgatcccaagcttcttctagaggtaccgcatgcgatAAGGA GGGGTGTTGCAGAGTGGATTATATGAATCT | KpnI | |
| ∆croIR2_RV | ggcccgatcccaagcttcttctagaggtaccgcatgcgat  ACGTCATCTGTCGTTATCATGTGTAGCGAACC | KpnI |  |
| pRE112_FW | atcgcatgcggtacctctagaagaagcttg | Confirming fragment insertion in pRE112 | |
| pRE112_RV | gagctctcccgggaattcatgcagttcac | Confirming fragment insertion in pRE112 | |

*Restriction enzyme sites are underlined

**Table S4:** Oligonucleotide primers used for the construction of chromosomally complemented strains.

| **Primer Name** | **Sequence*** | **Notes** |
| --- | --- | --- |
| Tn7-CroI_FW | gccttcgcgaggtaccTGTACCCGAGAGTAC  AAATGTAAGATAATCCCGTGCG | KpnI |
| Tn7-CroI_RV | ggctgcaggaattcctcgagTTACATACGGCG  CAGTTGTTGAAGCATCTCACG | XhoI |
| Tn7-CroR_FW | gccttcgcgaggtaccCGGGATTTCTAAAGA  AGAAGTTTGTACATTGATTAATGATA TAAGCCT | KpnI |
| Tn7-CroR_RV | ggctgcaggaattcctcgagTCAGTTATCCAGT  AATCTGAGCTCCATGCCGAGTTTTA CCGCATG | XhoI |
| PTn7R | CACAGCATAACTGGACTGATTTC | Confirming chromosomal insertion of mini-Tn7 elements |
| PglmS-down_Citro | GCACGTTGAGGAAGTCATTGC | Confirming chromosomal insertion of mini-Tn7 elements |
| Tn7-check_FW | tagttgggaactgggagggg | Confirming fragment insertion in mini-Tn7 |
| Tn7-check_RV | tccgaagttcctattctctagaaagt | Confirming fragment insertion in mini-Tn7 |

* Restriction enzyme sites are underlined

**Table S5:** Oligonucleotide primers used in this study for RT-qPCR.

| **Primer Name** | **Sequence** | **Notes** |
| --- | --- | --- |
| dnaQ_FW | GTCAGGCCCGCAAATTAC | Endogenous control |
| dnaQ_RV | TCTCCACCAGATCGAGACG | Endogenous control |
| recA_FW | TGCCACTACCTGGCTGAAAG | Endogenous control |
| recA_RV | CGTGGAGTCCTGGTTGTTGA | Endogenous control |
| eae_FW | GGTTAATCTGCAGAGCGGTAA | Intimin (Type III secretion system) |
| eae_RV | GAACGGTAATAAGAAGTCCAGTGAA | Intimin (Type III secretion system) |
| espA_FW | AATCACCGGCGCTTAACTCA | Translocator protein (Type III secretion system) |
| espA_RV | TTCACGCACAAAGCGAACTG | Translocator protein (Type III secretion system) |
| espB_FW | ATGTTCGCAGATCGCAGGAT | Effector protein (Type III secretion system) |
| espB_RV | TTACCCTGCTAAACGAGCCG | Effector protein (Type III secretion system) |
| tir_FW | TTGCATCGACCCAATGGTCA | Intimin receptor (Type III secretion system) |
| tir_RV | AGTGCTTTGGATACCCTGCC | Intimin receptor (Type III secretion system) |
| rpoS_FW | GAAGACACCACGCAGGATGA | RNA polymerase sigma factor (Stress Response) |
| rpoS_RV | CCGCTTCATAACCCAGCAGA | RNA polymerase sigma factor (Stress Response) |
| katE_FW | CATGACACCCGTGAATCCCA | Catalase HPII (Stress Response) |
| katE_RV | CCGTATCCGGCGATTTCAGA | Catalase HPII (Stress Response) |
| croI_FW | GCAGCCAGTCACCAGCTTTG | AHL Synthase (Quorum Sensing) |
| croI_RV | TGCGAACTGGAAGGTGGAGG | AHL Synthase (Quorum Sensing) |

# Table S6: MRM parameters optimized for the identification and quantification of AHLs.

Multiple-reaction monitoring (MRM) parameters used for AHL detection and quantification. MF, Molecular Formula; MW, Molecular Weight; Q1, Precursor Ion; Q3, Product Ion; Rt, Retention Time; Frag, Fragmentor; CE, Collision Energy.

| **Compound** | **MF** | **MW** | **Q1/Q3**  **quantification** | **Q1/Q3**  **confirmation** | **Rt (min)** | **Dwell** | **Frag (V)** |
| --- | --- | --- | --- | --- | --- | --- | --- |
| C4-HSL | C_8_H_13_NO_3_ | 171.0895 | 172.0/102 | 172/43 | 8.4 | 80 | 80 |
| C6-HSL | C10H17NO3 | 199.1208 | 200.1/102 | 200.1/71 | 12.0 | 80 | 80 |
| 3-oxo-  C6-HSL | C10H15NO4 | 213.1001 | 214.1/113 | 214.1/102 | 9.1 | 60 | 70 |
| 3-hydroxy-  C6-HSL | C10H17NO4 | 215.1158 | 216.1/102 | 216.1/69 | 8.0 | 60 | 80 |

| **Compound** | **CE (V)** | **Cell Accelerator (V)** |
| --- | --- | --- |
| C4-HSL | 5 | 7 |
| C6-HSL | 6 | 7 |
| 3-oxo-  C6-HSL | 5 | 7 |
| 3-hydroxy-  C6-HSL | 5 | 7 |


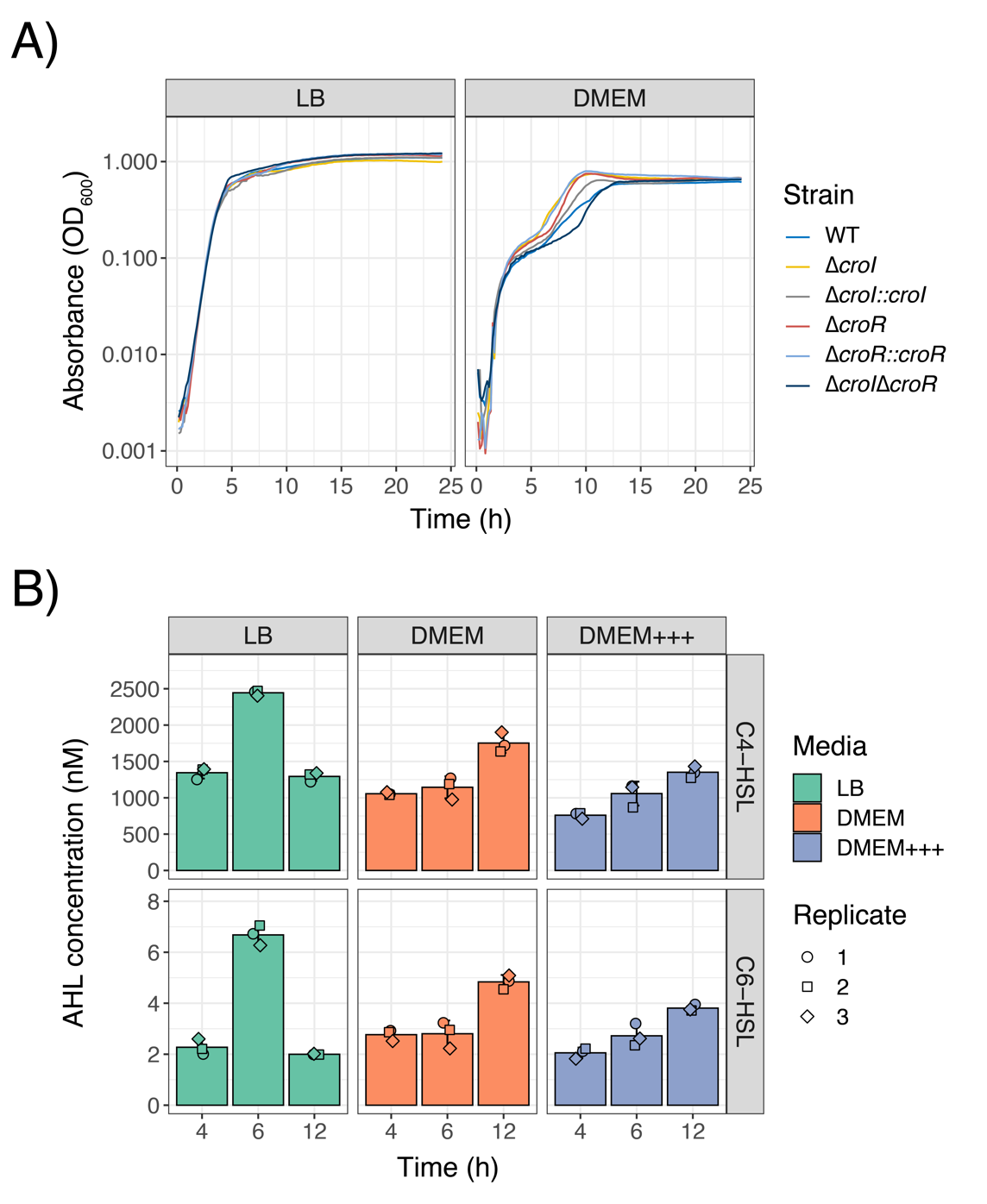


**Figure S1: In vitro growth dynamics of *C. rodentium* and AHL production profile across time.**

**A)** *In vitro* growth dynamics of *C. rodentium* WT DBS100, ∆*croI*, ∆*croR*, ∆*croI∆croR* and complemented strains. Strains were grown over the course of 24 hours in either LB or DMEM media at 37 °C with continuous shaking. The curves represent the average of three biological replicates measured in at least three technical replicates each. **B)** Accumulation of C4-HSL and C6-HSL produced by *C. rodentium* during growth in different culture media. Cell-free supernatants were collected and partially purified using ethyl acetate before analysis via LC-MS/MS. Trace amounts of 3-hydroxy-C6-HSL were also detected in all conditions tested (data not shown). Data represent the mean of 3 biological replicates *±* SD. LB, lysogeny broth; DMEM, Dulbecco’s Modified Eagle Medium; DMEM+++, Dulbecco’s Modified Eagle Medium supplemented with 10 % heat-inactivated fetal bovine serum, 1 % Non-Essential Amino Acids, and 1 % L-glutamine.


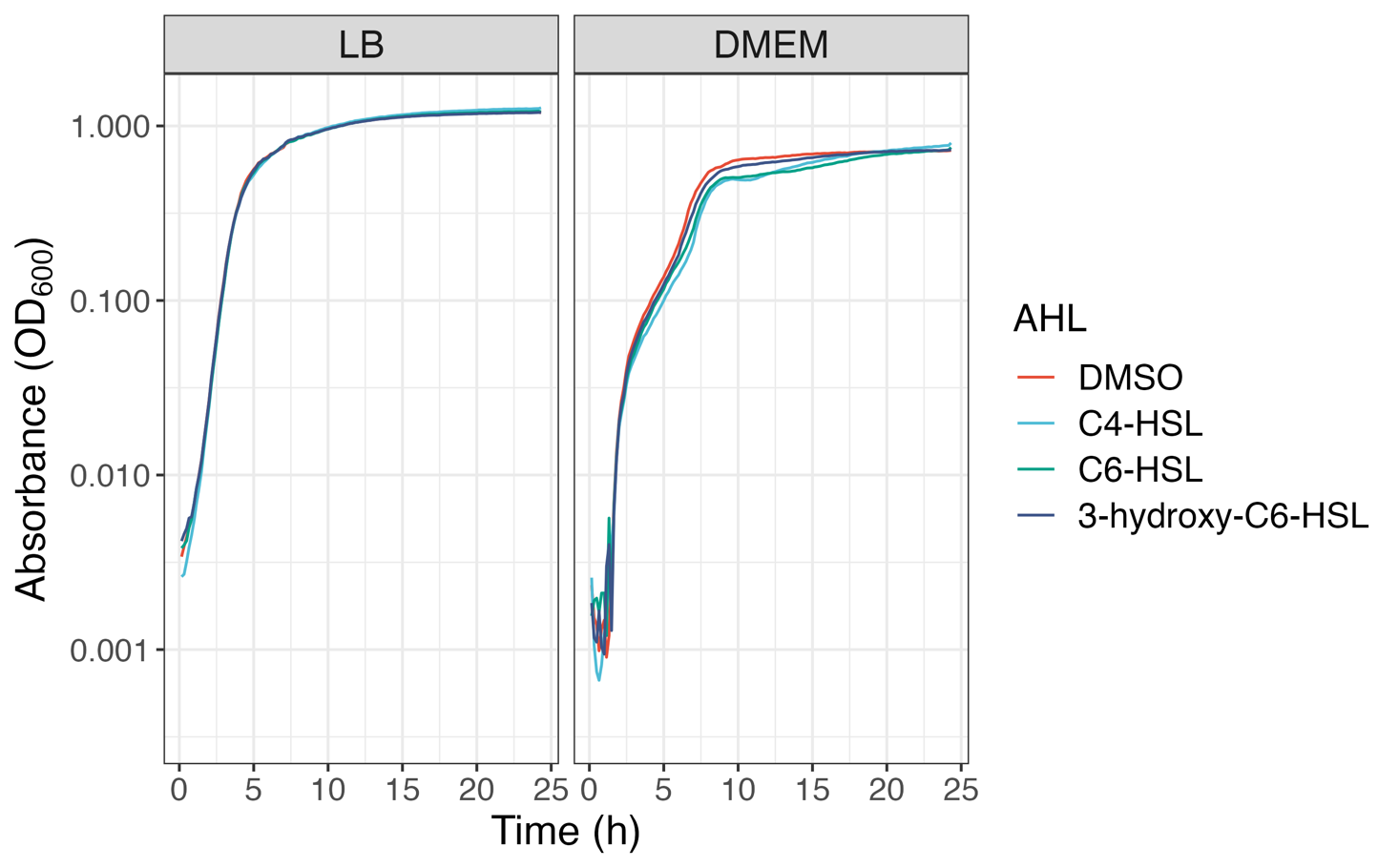


**Figure S2: Growth dynamics of WT *C. rodentium* supplemented with different AHLs.**

WT *C. rodentium* was grown in LB or DMEM media for 24 hours at 37 °C with continuous shaking. Cultures were supplemented with either 10 µM of C4-HSL, C6-HSL, 3-hydorxy-C6-HSL or DMSO as vehicle control, showing no impact of AHLs on growth of *C. rodentium*. Data represent the average of three independent biological replicates with three technical replicates each.


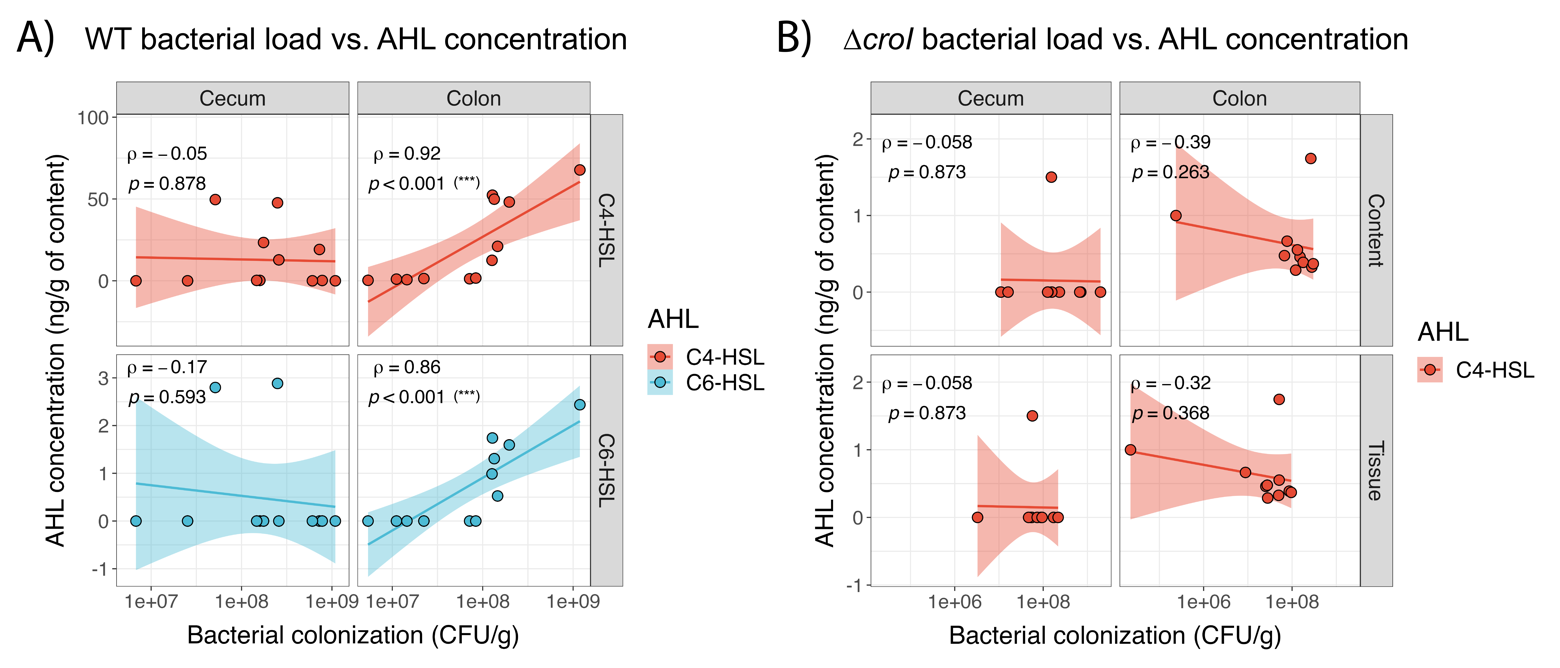


**Figure S3: *In vivo* AHL concentration measured in the colon correlates with *C. rodentium* burden.**

**A)** Spearman correlations between the detected C4-HSL and C6-HSL concentration and the CFU burden of WT *C. rodentium* in cecal and colonic lumenal content at day 7 post-infection. **B)** Spearman correlations of detected C4-HSL concentrations and the CFU burden of ∆*croI C. rodentium* in lumenal and tissue-associated subpopulations at the cecum and colon on day 7 post-infection. Rho (*ρ*) represents the Spearman correlation values; (*p*) represents the *p*-value. Line represents the linear regression; shaded area represents the 95 % confidence interval.


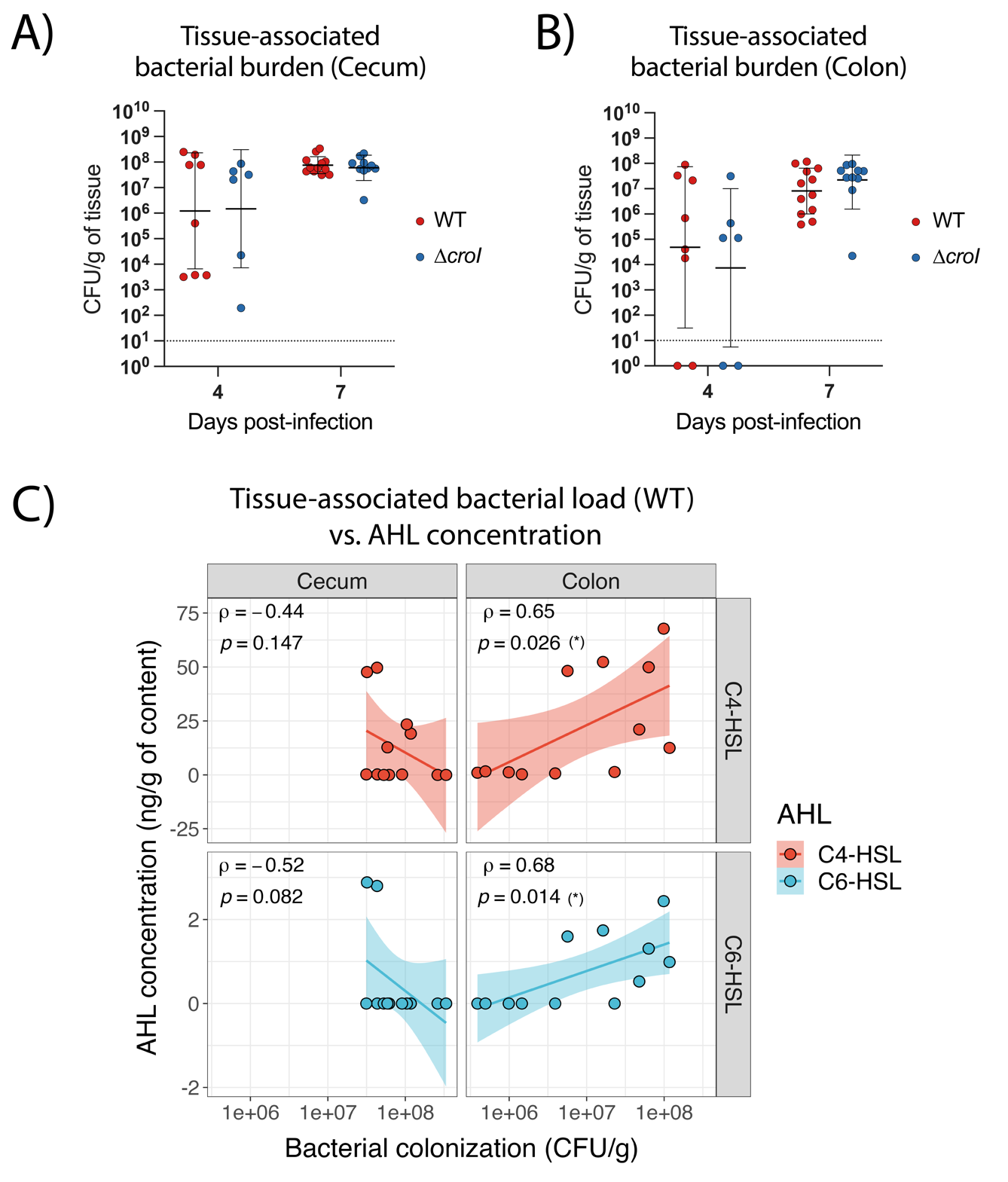


# Figure S4: Mucosal-associated CFU burden of *C. rodentium* during infection.

**A-B)** Tissue-associated burden of WT *C. rodentium* in the cecum and colon at day 4 and 7 post-infection (p.i.). Lines represent geometric mean *±* geometric standard deviation, with an N = 6-12 mice per group. The limit of detection is displayed as a dotted line. **C)** Spearman correlations between the detected C4-HSL and C6-HSL concentration and the tissue-associated burden of WT *C. rodentium* in the cecum and colon at day 7 post-infection. Rho (*ρ*) represents the Spearman correlation values; (*p*) represents the *p*-value. Line represents the linear regression; shaded area represents the 95 % confidence interval.


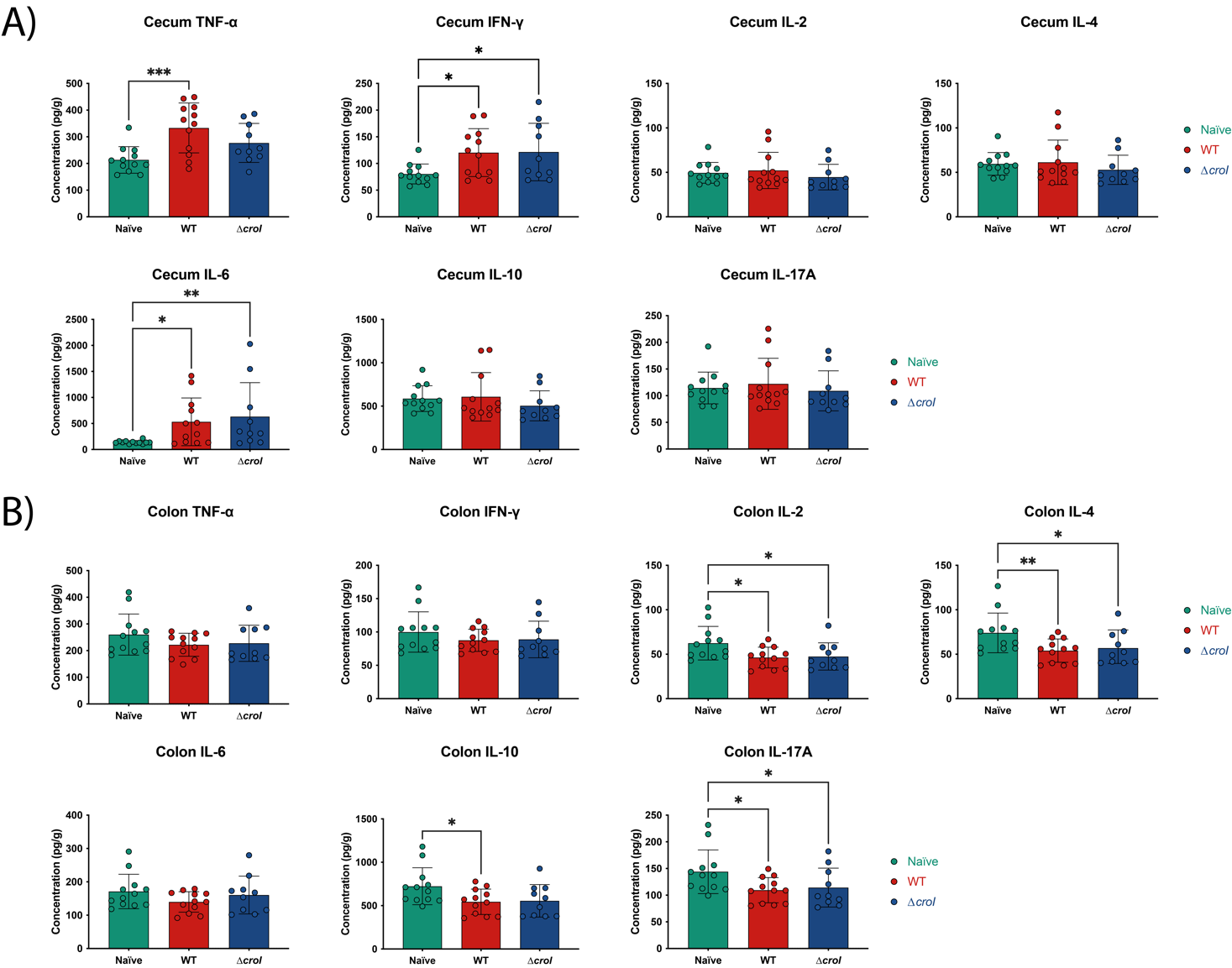


# Figure S5: Measurement of inflammatory cytokine markers in cecal and colonic tissues of uninfected and WT- and ∆*croI*-infected mice.

Tissues were collected at day 7 post infection and were measured using Cytokine Bead Array (CBA). Data represent the mean *±* SD with an N = 10-12. Statistical analysis was performed by using a Kruskal-Wallis test. TNF-*α*, Tumour Necrosis Factor alpha; IFN-*γ*, Interferon gamma;

IL-2, Interleukin-2; IL-4, Interleukin-4; IL-6, Interleukin-6; IL-10, Interleukin-10; IL-17, Interleukin-17.


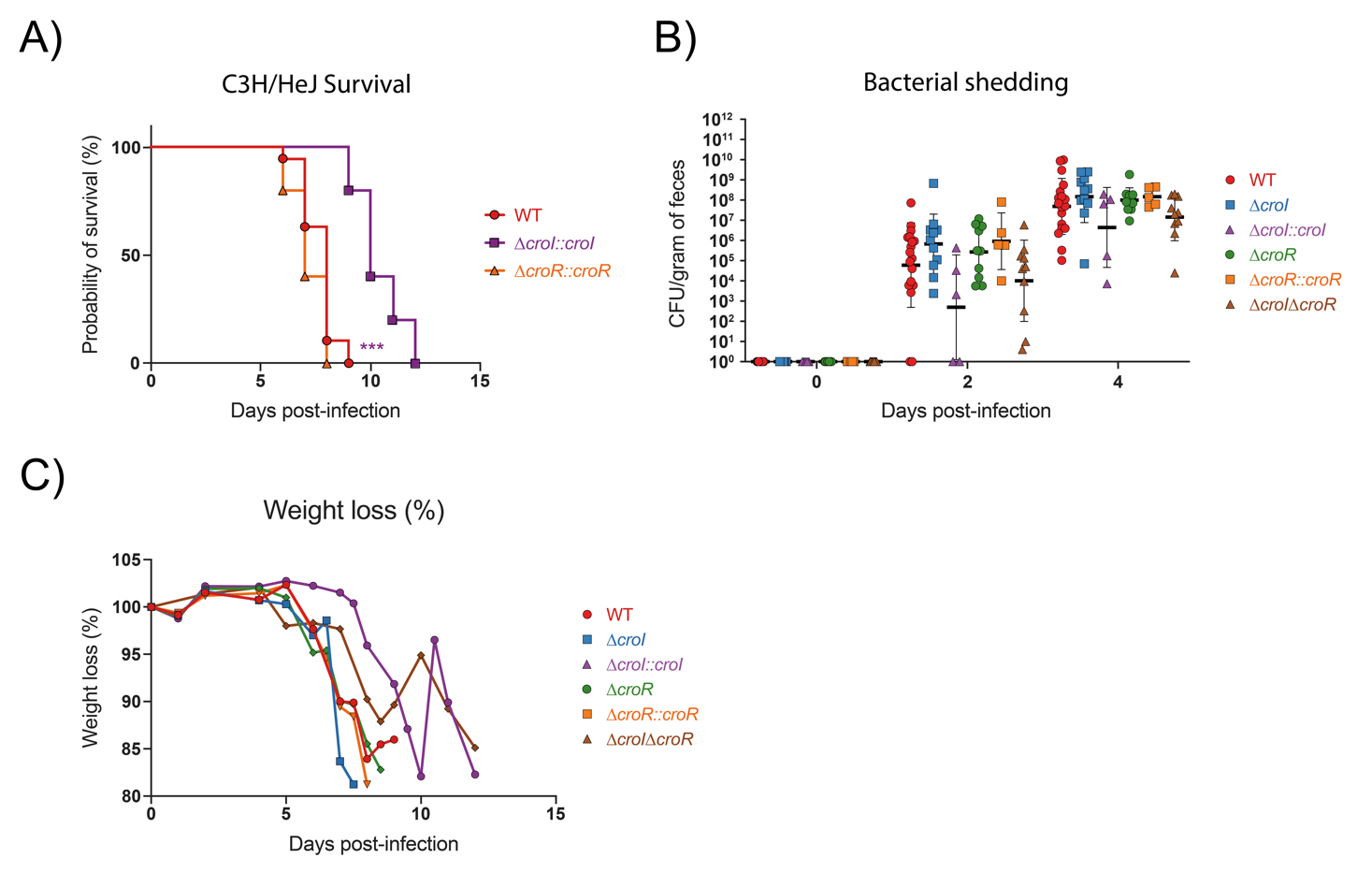


**Figure S6: Morbidity and mortality of susceptible C3H/HeJ mice infected with WT, ∆*croI,* ∆*croR* and complemented strains of *C. rodentium.***

**A)** Survival of C3H/HeJ mice infected with WT, ∆*croI::croI*, and ∆*croR::croR* complement strains. For WT, an N = 16 was used, while for each of the complement strains tested, an N = 5 was used instead. Statistics were calculated using a Gehan-Breslow-Wilcoxon test comparing each strain against WT *C. rodentium.* **B)** Fecal shedding of *C. rodentium* throughout the infection time course for all strains. **C)** Percentage of weight lost after infection with *C. rodentium*. The data represent the percentage lost compared to the initial weight prior to the infection.

References:

1. Schauer, D.B., and Falkow, S. (1993). The eae gene of Citrobacter freundii biotype 4280 is necessary for colonization in transmissible murine colonic hyperplasia. Infection and Immunity *61*, 4654–4661. 10.1128/iai.61.11.4654-4661.1993.

2. Ferrières, L., Hémery, G., Nham, T., Guérout, A.M., Mazel, D., Beloin, C., and Ghigo, J.M. (2010). Silent mischief: Bacteriophage Mu insertions contaminate products of Escherichia coli random mutagenesis performed using suicidal transposon delivery plasmids mobilized by broad-host-range RP4 conjugative machinery. Journal of Bacteriology *192*, 6418–6427. 10.1128/JB.00621-10.

3. Choi, K.H., and Schweizer, H.P. (2006). mini-Tn7 insertion in bacteria with single attTn7 sites: Example Pseudomonas aeruginosa. Nature Protocols *1*, 153–161. 10.1038/nprot.2006.24.

4. Edwards, R.A., Keller, L.H., and Schifferli, D.M. (1998). Improved allelic exchange vectors and their use to analyze 987P fimbria gene expression. Gene *207*, 149–157. 10.1016/S0378-1119(97)00619-7.

Unprocessed gels:

Profile of T3SS secreted proteins derived from the supernatants of WT, ∆*croI*, ∆*croR*, ∆*croI∆croR* and complemented strains of *C. rodentium* as shown on an SDS-PAGE gel stained with Coomassie Blue G250. Lanes that were irrelevant to this study were removed from the analysis and are presented in grayscale and with a transparency effect.


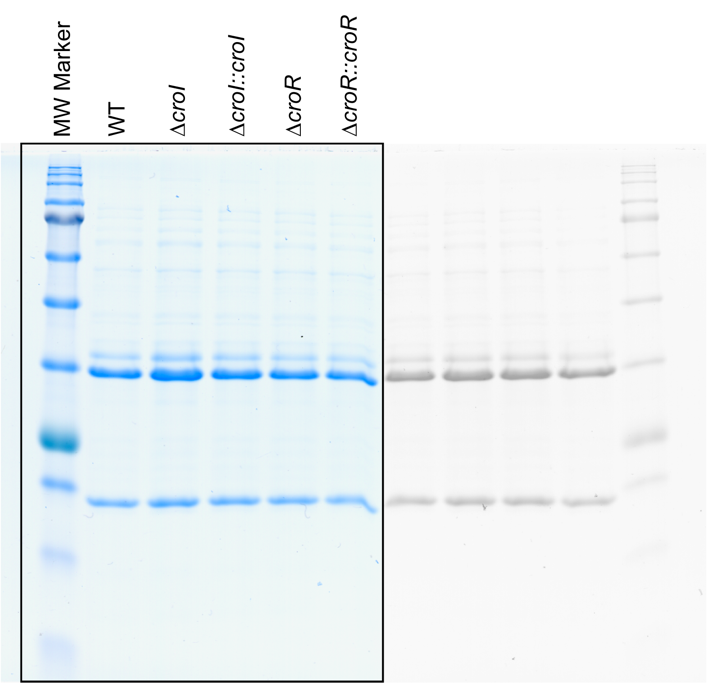


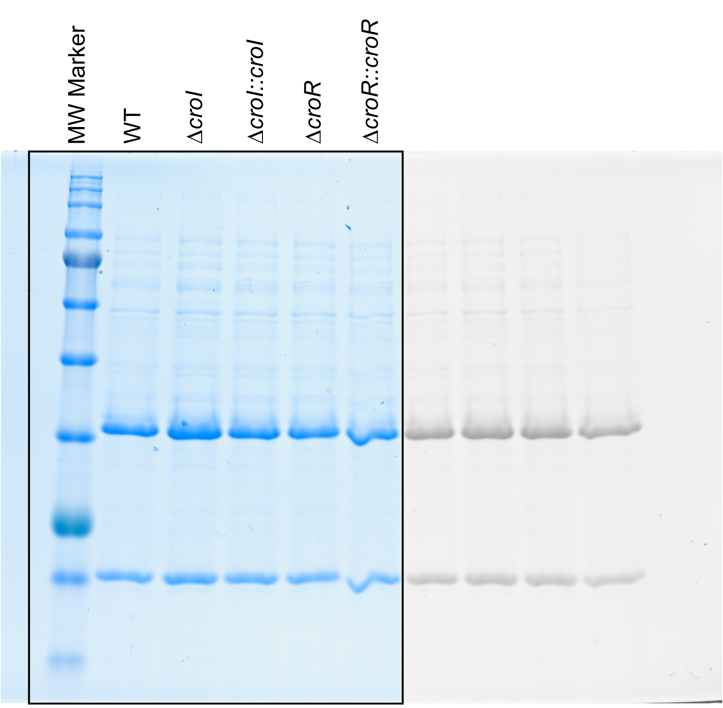


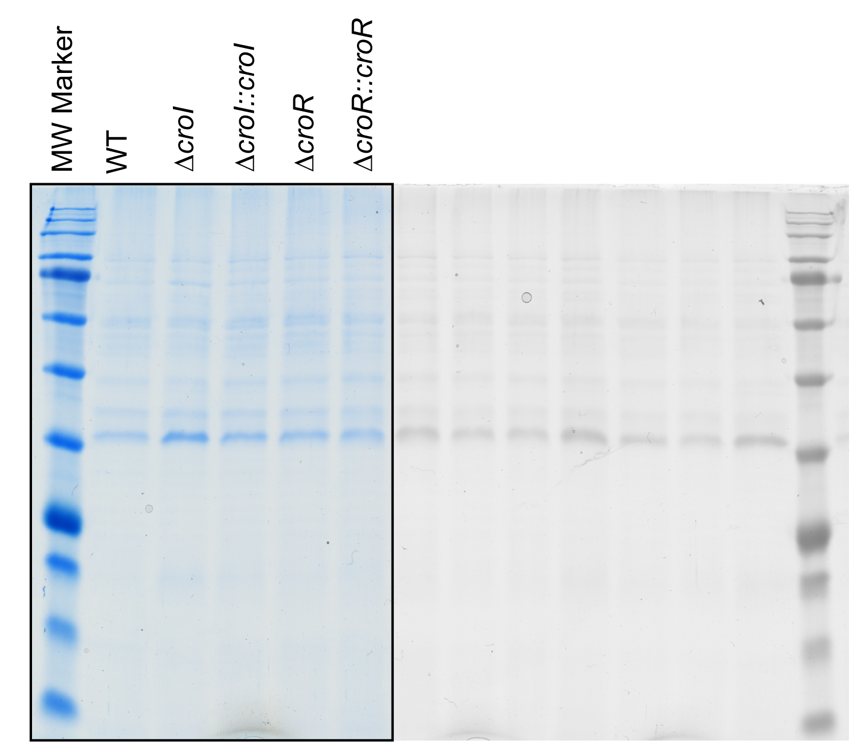


**
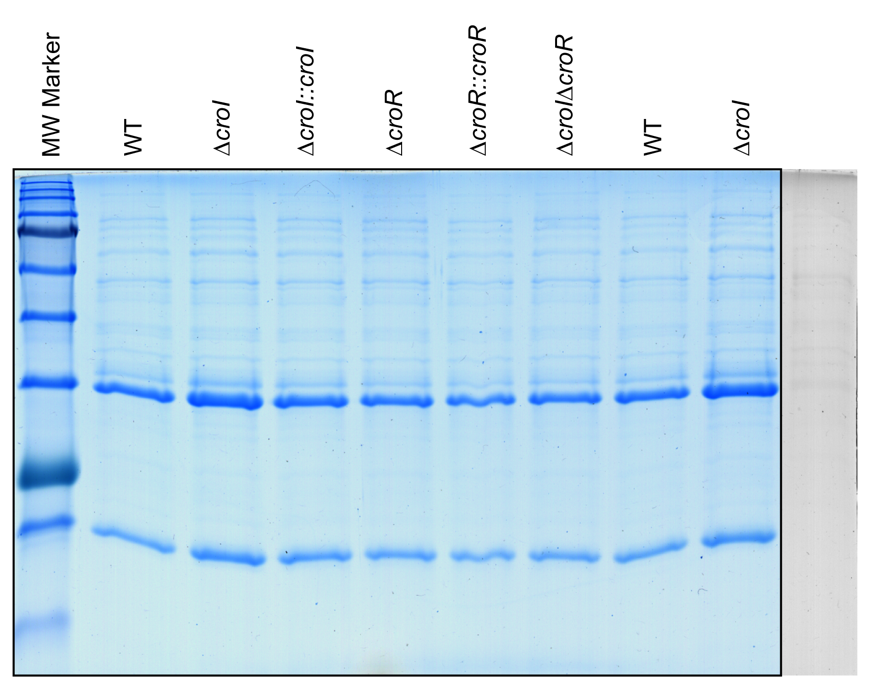
**

**
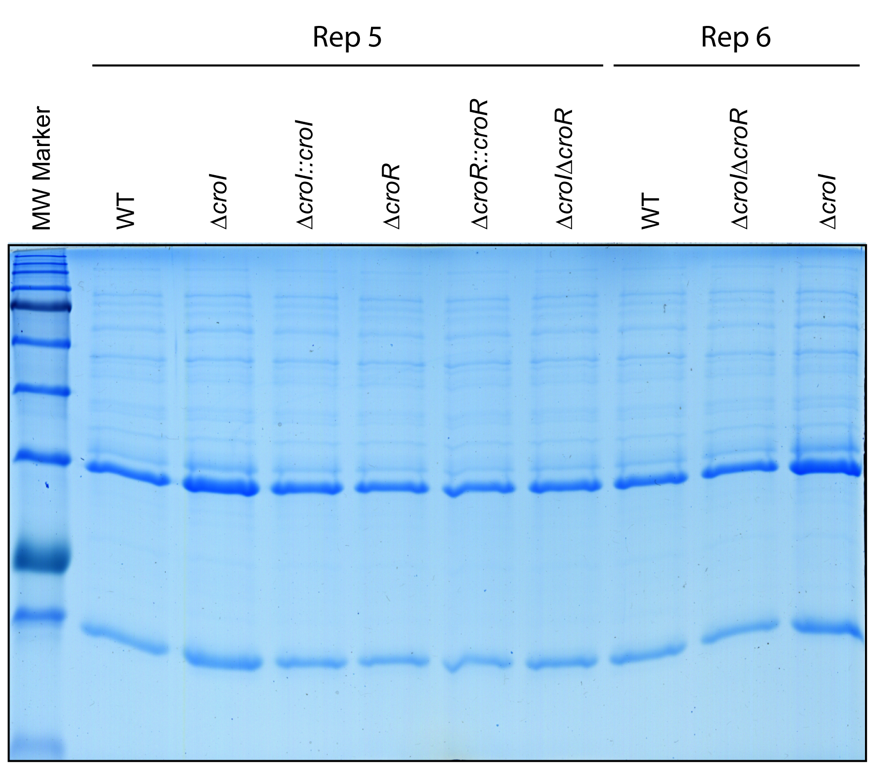
**


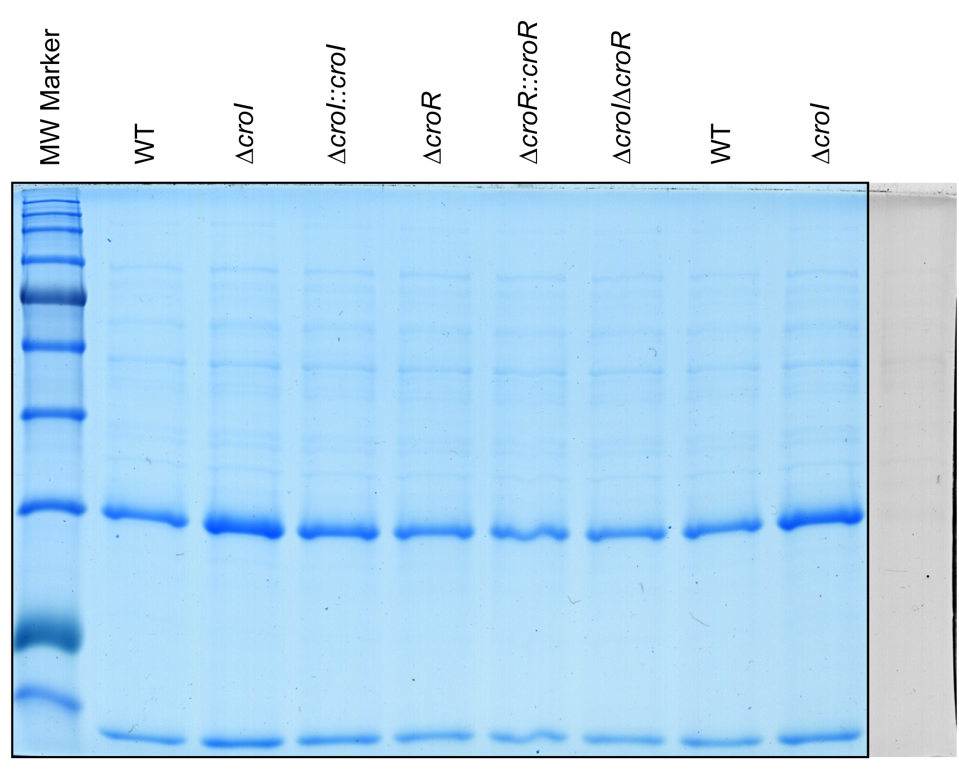

Supplement: Supplemental Material [file KGMI_A_2267189_SM9150.docx]
